# Disarming a molecular brake: cAMP-responsive element modulator deletion supercharges CAR-NK cells

**DOI:** 10.1038/s41392-025-02362-0

**Published:** 2025-08-28

**Authors:** Sudheendra Hebbar Subramanyam, Klaus Tenbrock

**Affiliations:** 1 Department of Pediatrics, Translational Pediatric Rheumatology and Immunology, Rheinisch-Westfälische Technische Hochschule University Hospital, Aachen, Germany; 2 Division of Pediatric Rheumatology, Department of Pediatrics, Inselspital, Bern University Hospital, University of Bern, Bern, Switzerland

**Keywords:** Molecular medicine, Tumour immunology

In a recent study published in *Nature*, Rafei and colleagues identified the transcription factor cAMP responsive element modulator (CREM) as an important regulator in natural killer (NK) cells that have been engineered with Chimeric antigen receptors (CARs). Their study demonstrated that CREM integrates signals from CAR activation and from interleukin-15 (IL-15) stimulation and serves as a molecular brake that limits CAR-NK functionality.^1^

CAR-NK cell therapy represents a rapidly advancing and highly promising strategy in cancer immunotherapy. CAR-NK cells are particularly well-suited for adoptive immunotherapy because they are able to recognize and eliminate cancer cells without dependence on major histocompatibility complex (MHC) compatibility. This capacity stems from their use of specialized receptors, such as NKG2D, which detect the absence of “self” markers and the presence of stress signals on malignant cells.^2^ In contrast to T-cell therapies, which often require meticulous human leukocyte antigen (HLA) matching to avoid complications like graft-versus-host disease (GvHD), NK cells can operate effectively across different donors and recipients. This MHC-independent targeting not only reduces the risk of GvHD but also allows the possibility to create “off-the-shelf” NK cell therapies, making them safer and more scalable than conventional T-cell approaches.^3,4^ Nevertheless, the efficacy of CAR-NK cells remains constrained by challenges such as cellular exhaustion, limited persistence, and suboptimal tumor infiltration. Elucidating the molecular mechanisms that govern CAR-NK cell function is therefore essential for enhancing their therapeutic potential.

Rafei and colleagues specifically showed that CREM functions as a transcriptional gatekeeper, restricting the cytotoxic capacity of CAR-NK cells against cancer cells. These findings demonstrate that CREM is a promising target to enhance the efficacy of CAR-NK cell therapies and open new avenues for advancing cancer treatment outcomes.

Interleukin-15 (IL-15) is a pivotal cytokine in NK cell biology, essential for their survival, proliferation, and cytotoxic function. Engineering NK cells to express IL-15 in the context of CAR-NK cell therapy further augments their persistence and antitumor activity, thereby enhancing the overall effectiveness of this innovative immunotherapy.^5^

Rafei et al. analyzed single-cell transcriptional profiles of CAR NK cells that were adoptively transfered into a mouse model of Raji lymphoma. This analysis revealed several differentially regulated genes, including CREM, whose expression coincided with peak antitumor activity and upregulation of cytotoxic molecules such as granulysin (GNLY), granzyme H (GZMH), granzyme B (GZMB), as well as genes associated with calcium signaling. Additionally, inhibitory receptors such as KIR2DL3, ADGRG1, and KLRG1 were upregulated, mirroring the exhausted phenotype observed in T cells. Notably, a positive correlation was found between CREM expression and IL-15 signaling activity across all NK cell cycle stages, suggesting that CREM dynamically regulates NK cell responses.

To further delineate the upstream regulation of CREM, the authors employed various engineered CAR constructs targeting CD70, some with intact signaling domains and others with mutated or absent intracellular motifs. CAR constructs capable of activating immunoreceptor tyrosine-based activation motifs (ITAMs) induced robust CREM expression upon antigen engagement. Parallel experiments established IL-15 as the most potent inducer of CREM.CREM induction followed a dose-dependent manner. Moreover, simultaneous IL-15 stimulation and CAR activation-induced CREM expression synergistically. Given that CREM exists in multiple isoforms acting as either transcriptional activators or repressors, the authors showed that CAR-NK cells predominantly express repressor isoforms upon activation. Mass cytometry further revealed increased expression of both activation and inhibitory markers in CREM-high NK cells, reinforcing the association of CREM with activation-induced exhaustion.

Delving deeper into the molecular mechanisms, the authors identified the protein kinase A (PKA)-CREB pathway as a key upstream regulator of CREM. CREB phosphorylation, triggered by both CAR signaling and IL-15 stimulation via activation of PKA, led to increased CREM transcription. Pharmacological inhibition of PKA or calcium chelation prevented CREB phosphorylation and inhibited expression of CREM, underscoring the importance of the PKA-CREB pathway in immune checkpoint regulation (Fig. 1). Additional experiments highlighted the role of IL-15 in activating STAT3 and STAT5. While only STAT5 directly bound to the CREM promoter, deletion of either STAT3 or STAT5A/B diminished CREM expression, though not as markedly as PKA-CREB inhibition. These results indicate that CREM is regulated by both canonical cytokine signaling (STAT5) and secondary messenger pathways (PKA-CREB), with the latter exerting a more dominant influence.

**Fig. 1:**
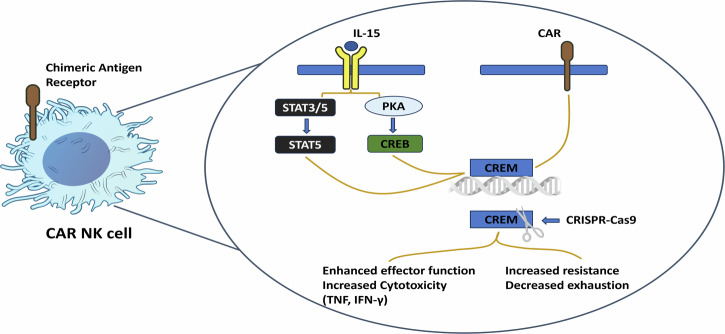
CREM as a regulatory checkpoint of CAR and IL-15 signaling in NK cells. This schematic illustrates the dual function of CREM in CAR-NK cells through two primary pathways: IL-15 signaling and CAR activation. Activation by IL-15 stimulates PKA-CREB pathway and/or STAT5, whereas CAR stimulation initiates ITAM signaling; both pathways ultimately leads to the induction of CREM. CRISPR-Cas9 deletion of CREM results in increased effector function with enhanced expression of TNF and IFN-γ and heightened resistance to tumor-induced immunosuppression, pinpointing the function of CREM as a potential intracellular regulator of CAR-NK cell function

Subsequent experiments assessed the functional impact of CREM deletion in CAR-NK cells using CRISPR-Cas9. The knockout of CREM (KO) strikingly increased the cytotoxicity of CAR-NK cells against various tumor targets in vitro, including renal carcinoma, lymphoma, pancreatic, and ovarian cancer cell lines. Upon repeated tumor challenges, CREM KO cells exhibited improved persistence and resistance, key attributes for sustained in vivo responses. CREM deletion also increased the expression of interferon-γ (IFN-γ) and tumor necrosis factor, further supporting CREM’s role as a suppressive checkpoint (Fig. 1). In vivo, CREM KO CAR70-IL-15 NK cells achieved superior tumor control and prolonged survival in a Raji lymphoma model, with similar benefits observed in breast and pancreatic cancer models, highlighting the enhanced functionality of CREM-deficient CAR-NK cells.

Subsequent investigation using ATAC-seq and ChIP-seq demonstrated that CREM occupies regulatory regions of genes involved in immune function and suppression, underscoring its pivotal role in orchestrating both activation and inhibitory programs. CREM KO cells exhibited altered chromatin accessibility at regulatory regions, favoring transcriptional profiles associated with effector function over exhaustion.

In summary, this study identifies CREM as a dynamic molecular brake on NK cell function within the context of CAR and IL-15 signaling. By elucidating the upstream pathways that regulate CREM expression and its downstream gene networks, the authors provide a roadmap for optimizing NK cell-based immunotherapies. Targeting CREM presents a novel strategy to amplify antitumor responses without compromising safety, potentially enabling more durable and effective cancer treatments.

## References

[1] Rafei, H. et al. CREM is a regulatory checkpoint of CAR and IL-15 signalling in NK cells. *Nature*10.1038/s41586-025-09087-8 (2025). 40468083 10.1038/s41586-025-09087-8 PMC12286855

[2] Laskowski, T. J., Biederstädt, A. & Rezvani, K. Natural killer cells in antitumour adoptive cell immunotherapy. *Nat. Rev. Cancer* **22**, 557–575 (2022). 35879429 10.1038/s41568-022-00491-0 PMC9309992

[3] Chen, S., Zhu, H. & Jounaidi, Y. Comprehensive snapshots of natural killer cells functions, signaling, molecular mechanisms and clinical utilization. *Signal Transduct. Target Ther.* **9**, 302 (2024). 39511139 10.1038/s41392-024-02005-w PMC11544004

[4] Moazzeni, A., Kheirandish, M., Khamisipour, G., Rahbarizadeh, F. & Pourfathollah, A. A. Leukoreduction filter derived NK cells offer a promising source for off the shelf CAR NK cell immunotherapy. *Sci. Rep.* **15**, 12755 (2025). 40223011 10.1038/s41598-025-97584-1 PMC11994799

[5] Jiang, Q. et al. Robust differentiation of NK cells from MSLN.CAR-IL-15-engineered human iPSCs with enhanced antitumor efficacy against solid tumors. *Sci. Adv.* **11**, eadt9932 (2025). 40315330 10.1126/sciadv.adt9932 PMC12047432

